# Spatial and activity‐dependent catecholamine release in rat adrenal medulla under native neuronal stimulation

**DOI:** 10.14814/phy2.12898

**Published:** 2016-09-05

**Authors:** Kyle Wolf, Georgy Zarkua, Shyue‐An Chan, Arun Sridhar, Corey Smith

**Affiliations:** ^1^ Department of Physiology and Biophysics Case Western Reserve University Cleveland Ohio; ^2^ Bioelectronics Research and Development GlaxoSmithKline Stevenage United Kingdom

**Keywords:** Adrenal medulla, catecholamine, stress, sympathetic

## Abstract

Neuroendocrine chromaffin cells of the adrenal medulla in rat receive excitatory synaptic input through anterior and posterior divisions of the sympathetic splanchnic nerve. Upon synaptic stimulation, the adrenal medulla releases the catecholamines, epinephrine, and norepinephrine into the suprarenal vein for circulation throughout the body. Under sympathetic tone, catecholamine release is modest. However, upon activation of the sympathoadrenal stress reflex, and increased splanchnic firing, adrenal catecholamine output increases dramatically. Moreover, specific stressors can preferentially increase release of either epinephrine (i.e., hypoglycemia) or norepinephrine (i.e., cold stress). The mechanism for this stressor‐dependent segregated release of catecholamine species is not yet fully understood. We tested the hypothesis that stimulation of either division of the splanchnic selects for epinephrine over norepinephrine release. We introduce an ex vivo rat preparation that maintains native splanchnic innervation of the adrenal gland and we document experimental advantages and limitations of this preparation. We utilize fast scanning cyclic voltammetry to detect release of both epinephrine and norepinephrine from the adrenal medulla, and report that epinephrine and norepinephrine release are regulated spatially and in a frequency‐dependent manner. We provide data to show that epinephrine is secreted preferentially from the periphery of the medulla and exhibits a higher threshold and steeper stimulus‐secretion function than norepinephrine. Elevated stimulation of the whole nerve specifically enhances epinephrine release from the peripheral medulla. Our data further show that elimination of either division from stimulation greatly attenuated epinephrine release under elevated stimulation, while either division alone can largely support norepinephrine release.

## Introduction

The adrenal medulla is composed of a highly vascularized cluster of neuroendocrine adrenal chromaffin cells. Upon stimulation through the sympathetic splanchnic nerve, the catecholamines, epinephrine, and norepinephrine are released from adrenal chromaffin cells into the circulation for delivery throughout the periphery where they regulate diverse physiological systems (Habib et al. 2001). Anatomically, the splanchnic–adrenal pathway is somewhat unconventional compared to the rest of the sympathetic nervous system. Most preganglionic sympathetic neurons exit the spinal cord and form a cholinergic synapse in the paravertebral sympathetic chain ganglia or paravertebral ganglia (celiac ganglia, superior and inferior mesenteric ganglia). The efferent postganglionic neurons then innervate target organs, releasing norepinephrine at a rate dictated by sympathetic firing (Carmichael 1986). However, this is not the case with the splanchnic innervation of the adrenal gland. In this instance, the spinal sympathetic efferents exit the spinal cord as the splanchnic nerve and pass through the sympathetic chain ganglia and continue to the adrenal medulla where they then form a cholinergic synapse with the adrenal chromaffin cells. Upon stimulation, the chromaffin cells then release catecholamines into the circulation. Thus, the adrenal medulla is considered a displaced sympathetic ganglion and the chromaffin cells resemble postganglionic sympathetic neurons. Three notable characteristics of the chromaffin cells separate them from postganglionic neurons. Chromaffin cells are morphologically very simple. Unlike postganglionic neurons that form axons and secrete norepinephrine onto their target organ, chromaffin cells have no dendritic arbors or axonal outgrowths; they are conical in shape (Carmichael 1986). Second, chromaffin cells do not secrete catecholamines in close proximity to their specific target organ, they release catecholamines into the general circulation to affect multiple, distant target organs. Finally, while postganglionic sympathetic neurons release norepinephrine, chromaffin cells release either norepinephrine or epinephrine. Thus, the adrenal medulla is the exclusive source of epinephrine for circulation throughout the periphery. In chromaffin cells, norepinephrine versus epinephrine release is determined on the cellular level by the expression of phenylethanolamine N‐methyltransferase (PNMT), the terminal enzyme in epinephrine synthesis. Thus, chromaffin cells that express PNMT secrete mainly epinephrine and those negative for PNMT expression release norepinephrine (Goldstein et al. 1971).

Early studies of the sympathoadrenal stress response demonstrated a stressor‐dependent preferential release of epinephrine or norepinephrine from the adrenal medulla to initiate the appropriate physiological response to the given stressor (Coupland 1958; Klevans and Gebber 1970; Vollmer et al. 1992; Vollmer 1996; Goldstein 2010; Kvetnansky et al. 2013). It has been demonstrated that with splanchnic nerve stimulation, there is a frequency‐dependent shift in catecholamine release, in which norepinephrine and epinephrine release exhibit specific frequency dependence (Damase‐Michel et al. 1993). Thus, there must be a differential stimulation mechanism for norepinephrine‐ versus epinephrine‐secreting cells. The splanchnic nerve bifurcates into two divisions, anterior and posterior branches, before innervating the adrenal gland (Celler and Schramm 1981). There is also evidence for the differential innervation of epinephrine and norepinephrine‐secreting cell types by histologically and electrophysiologically distinguishable nerve fibers (Edwards et al. 1996; Cao and Morrison 2001), raising the intriguing hypothesis that either division of the splanchnic represents specific innervation of either epinephrine‐ or norepinephrine‐secreting chromaffin cells. However, the hypothetical physiological role for these two divisions of the splanchnic nerve on epinephrine versus norepinephrine release has not been tested. We addressed this hypothesis in a novel rat ex vivo splanchnic–adrenal experimental system. The innervating splanchnic nerve was stimulated to evoke catecholamine release from a hemisected adrenal gland. We then utilized fast scanning cyclic voltammetry (FSCV) (Kawagoe et al. 1991; Leszczyszyn et al. 1991) to identify and measure evoked epinephrine and norepinephrine release. Next, we generated a spatial map of epinephrine and norepinephrine release from the adrenal medulla under varied neuronal stimulation frequencies. Finally, we tested the activity of both branches of the splanchnic to control either epinephrine or norepinephrine release and we report here that there is a distinct central versus peripheral spatial distribution of their release within the adrenal medulla. Moreover, while there is no measurable correlation between which splanchnic branch is stimulated and epinephrine versus norepinephrine release, we report an overall increased stimulus threshold for epinephrine release over norepinephrine release. Increased splanchnic stimulation specifically increases epinephrine release from the peripheral adrenal medulla. This elevated epinephrine release requires concomitant excitation of both branches of the splanchnic. Stimulation of either branch singly fails to show enhanced epinephrine release. However, excitation of either single branch is largely sufficient to support norepinephrine release.

## Materials and Methods

### Ethical approval

Animal care and use was in accordance with National Institutes of the Health and Case Western Reserve University institutional guidelines (United States Federal welfare assurance number #A3145‐01). All protocols were approved by the Institutional Animal Care and Usage Committee (IACUC) and are in accordance with the 2013 American Veterinary Medical Association guidelines for animal euthanasia.

### Chemicals

All chemicals were purchased from Sigma‐Aldrich (St. Louis, MO) and used as received unless otherwise specified. Epinephrine was obtained as l(−)Epinephrine from MP Biomedicals, LLC (Solon, OH). Electrochemical and ex vivo experiments were carried out in Tris (tris(hydroxymethyl) aminomethane)‐buffered saline (TBS; 132 mmol/L NaCl, 40 mmol/L Tris, 11.2 mmol/L Glucose, 4.2 mmol/L KCl, 2 mmol/L CaCl_2_, 0.7 mmol/L MgCl_2_) at pH 7.4. In vitro FSCV control experiments were conducted in TBS with epinephrine (Epi) or norepinephrine (NE) added as indicated in the text. All solutions were made from double deionized water >17.5 MΩ cm.

### Ex vivo preparation

Sprague Dawley rats (225–275 g, Charles River Laboratories, Raleigh, NC) were housed in the Animal Resource Center of Case Western Reserve University and were provided with food and water ad libitum. For tissue harvest, rats were deeply anesthetized with isoflurane inhalation and euthanized by decapitation and bilateral pneumothorax. Anesthesia was determined by monitoring the rat until completely unresponsive and breathing ceased. The peritoneum was opened and superfused with an ice‐cold low‐calcium physiological saline of the following composition (in mmol/L): 150 NaCl, 10 HEPES‐H, 10 Glucose, 2.8 KCl, 4.3 MgCl_2_, 0.5 CaCl_2_, pH to 7.2. The back wall of the peritoneum was rapidly dissected and isolated. This section of the wall extends between approximately vertebrae T1 and L5 and extends laterally to include the kidneys and adrenal glands. While all the viscera in the peritoneal cavity were removed, the kidneys and adrenal glands, and their associated vessels and nervous tissue in the retroperitoneal region, were preserved. The preparation was pinned out on a silicone elastomer substrate and the bath solution changed to a TRIS‐buffered saline (TBS) as described above. All recordings were performed at 23–25°C and within 1 h after animal termination.

Most rats presented a readily‐observable discrete bifurcation in the splanchnic nerve between sympathetic chain ganglion and the innervation of the adrenal gland. Previous anatomical studies of the rat splanchnic described heterogeneity where approximately 30% of rats did not exhibit two divisions in the splanchnic (Celler and Schramm 1981). We did not find this heterogeneity, but on occasion (approximately 15–20% of rats, by empirical observations), the divisions of the splanchnic were closely associated and not readily separable without damaging the nerves. In these instances, the animal was used for whole nerve recording only. Before recording, the adrenal gland was hemisected to expose the adrenal medulla. One carbon fiber was then placed at the periphery of the adrenal medulla, while another was placed in the center of the medulla. The positions of recording sites were recorded as central versus peripheral medulla. Stimulating electrodes and recording carbon fiber electrodes were positioned with the aid of a 40× stereo microscope (AmScope, Irvine, CA). FSCV experiments consisted of a 60‐sec relaxation period for the carbon fiber in the bath followed by electrical neuronal stimulation. The electrical stimulation was carried out by driving a stimulus isolator (A356, WPI, Sarasota, FL) running in constant current mode. Stimuli were delivered to the nerve through either a platinum/iridium parallel bipolar electrode (FHC, Bowdoin, ME) or a multipole cuff electrode (CorTec; Freiburg Germany) as 10 *μ*sec square bipolar pulses at a constant current of 200 *μ*A. Pulse trains were delivered at a frequency of 1 Hz, 5 Hz, or 10 Hz for 60 sec as described in the text. Both bipolar parallel and cuff electrodes limit leakage of the current and prevent stimulation of adjacent nerves compared to stimulation through unipolar electrodes and tissue grounds. Each preparation was stimulated with only a single frequency, providing a single recording. Catecholamine release was then measured at 180 sec poststimulation.

### Electrochemical electrode preparation

**Figure 1:**
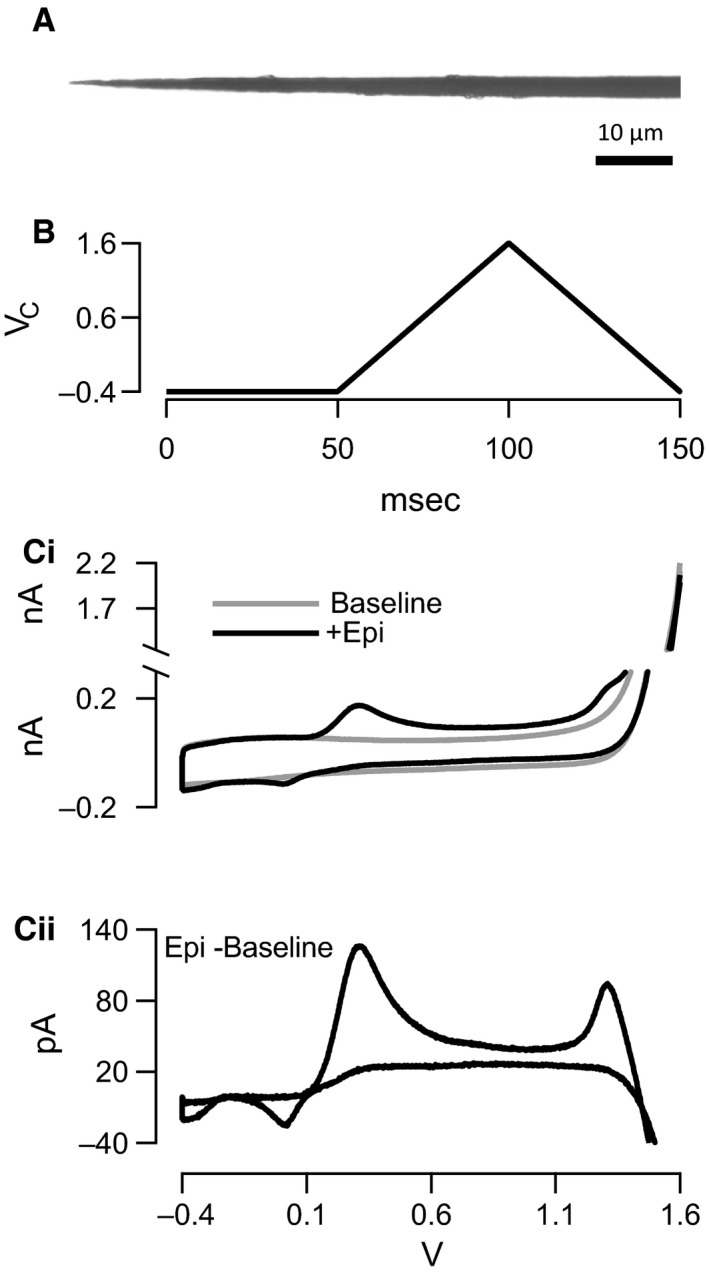
Flame‐etched carbon fiber fast scanning cyclic voltammetry. (A) Carbon fiber electrodes were used for all fast scanning cyclic voltammetry (FSCV) recordings. The fibers are 5 *μ*m in diameter and insulated with a parylene coating. Prior to use, each fiber was flame‐etched to provide a sharp point of uninsulated carbon surface and uniform surface area. (B) The scanning command potential for FSCV recordings is shown. The scan begins with a 50‐msec hold at −0.4 V to attract positively charged catecholamines to the electrode tip for detection. The scan then ramps from −0.4 V to 1.6 V and back at 40 V/sec, covering the oxidation and reduction potentials for epinephrine (Epi) and norepinephrine (NE). (Ci) Resulting voltammograms for background largely capacitative currents in Tris‐buffered saline (gray) and the same solution containing 250 *μ*mol/L Epi (black) are shown. (Cii) The subtraction of background current recorded in Tris‐buffered saline from that containing the Epi is provided and represents the Epi‐specific oxidation and reduction currents.

All electrochemical experiments were carried out with commercially available 5‐*μ*m diameter parylene‐insulated carbon fiber electrodes (CFE‐2, ALA Scientific, Farmingdale, NY). Care was taken to utilize electrodes of equivalent length throughout this study. Conventional carbon fiber amperometry or voltammetry utilizes blunt‐end electrodes, generated by a transverse cut of the tip with a scalpel blade. While this approach is simple and provides excellent results for amperometric assays of quantal catecholamine release, it did not provide low‐noise, fast‐response probes of consistent sensitivity needed for the fast scanning cyclic voltammetry utilized in this study. Therefore, we adopted a flame‐etch strategy to provide low‐noise, consistent fibers. Before experiments, electrodes were flame etched to remove insulation from the tip and to provide a reproducible tapered tip geometry. Flame etching was performed by submerging the carbon fiber electrode in a water bath with only a very short length extending above the surface. The tip was then flamed with an isobutane torch for 3–5 sec. Carbon fibers were inspected under a 40× stereo microscope to ensure proper tip geometry and removal of the parylene insulation from the tip (Fig. 1A).

### Data acquisition

Fast scanning cyclic voltammetry (FSCV) utilizes a dynamic command potential to oxidize and reduce molecules at the electrode tip. In our implementation of FSCV, the electrode was held at −0.4 V for 50 msec, ramped to 1.6 V, and then back to −0.4 V versus a Ag/AgCl bath ground at 40 V/sec (Fig. 1B). This waveform was generated and resulting digitized signal recorded through software custom written in Igor Pro (Wavemetrics, Lake Oswego, OR) controlling a Dagan ChemClamp amplifier fitted with a 1 MΩ resistive feedback head stage (Minneapolis, MN). Data were filtered at 5 kHz through an analog 2‐pole Bessel filter prior to digitization at 20 kHz through a HEKA ITC‐1600 analog/digital converter (HEKA Instruments, Hollister, MA). Under the scanning parameters utilized in this study, both epinephrine and norepinephrine exhibit a primary oxidation potential at approximately 300 mV while epinephrine, a secondary amine, exhibits a secondary oxidation potential at approximately 1.3 V.

## Results

### In vitro fast scanning cyclic voltammetry of epinephrine versus norepinephrine

We adapted a fast scanning cyclic voltammetry (FSCV) method for measuring separately the release of epinephrine (Epi) and norepinephrine (NE) under native neuronal stimulation. FSCV has been used to qualitatively measure release of catecholamine species from isolated bovine chromaffin cells (Pihel et al. 1994) and for the detection of bulk catecholamine release from mouse adrenal slices (Walsh et al. 2011). Here, we extend this technique by calibrating signals against standard solutions and by separating signal characteristic for catecholamine species in an ex vivo, intact splanchnic–adrenal experimental preparation. In order to perform quantitative FSCV to measure epinephrine versus norepinephrine, we employed flame‐etched carbon fiber electrodes (Fig. 1A, see Materials and Methods) and a command potential as described in Figure 1B. Resulting baseline currents recorded in Tris‐buffered saline (TBS) were largely nonspecific and represent capacitative charging of the fiber, oxidation/reduction of the fiber conductive surface, and some oxidation/reduction of the bath solution. However, when solutions were supplemented with Epi or NE, additional specific components emerged within the voltammogram (Fig. 1Ci) and after baseline background subtraction, the characteristic oxidation/reduction profile for NE and Epi were readily observable (see Fig. 1Cii for an Epi subtraction example).

**Figure 2:**
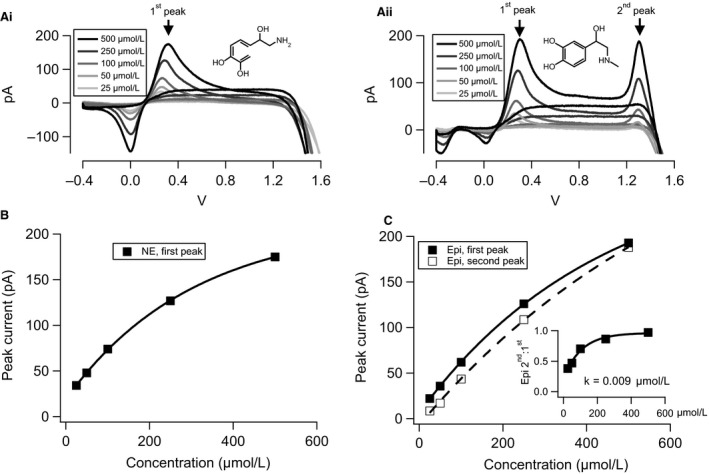
Current‐based calibration of the fast scanning cyclic voltammetry (FSCV) voltammograms. (Ai) Voltammograms were recorded and background subtracted as described in Figure 1. Panel Ai shows such example voltammograms for Tris‐buffered saline supplemented with norepinephrine (NE) over a range of concentrations relevant to the biological context. A single primary (“first”) oxidation current peak is observed for NE. (Aii) An equivalent set of FSCV voltammograms is provided for epinephrine (Epi)‐supplemented Tris‐buffered saline and display both a primary (“first”) and a secondary (“second”) oxidation peak current. The second peak is diagnostic for Epi. (B) The magnitude of the first peak in the NE calibration voltammogram set follows an exponential function depending on NE concentration. (C) Both the primary and secondary oxidation peaks for Epi follow exponential dependencies on concentration (Inset). The ratio of the magnitude of the second peak to the first peak in the Epi voltammogram follows an exponential function (reaction constant = 0.009 (*μ*mol/L)^−1^) and may be used to assign relative release of Epi to NE as described in the text.

We measured specific oxidation profiles for both NE (Fig. 2Ai) and Epi (Fig. 2Aii) in TBS, supplemented with either catecholamine at 25, 50, 100, 250, or 500 *μ*mol/L. NE is a primary catecholamine and exhibits a single oxidation potential and a single reduction potential as it undergoes oxidation to a quinone and reduction back to norepinephrine. Epinephrine undergoes the same primary oxidation reaction and thus exhibits the same primary oxidation potential as NE. However, Epi oxidation exhibits a secondary current peak at approximately 1.3 V and a second reduction potential at approximately −0.4 V (Chen and Peng 2003). Thus, the primary (first) peak provides a measure of total catecholamine (NE + Epi), while the second peak provides an Epi‐specific signal.

**Figure 3:**
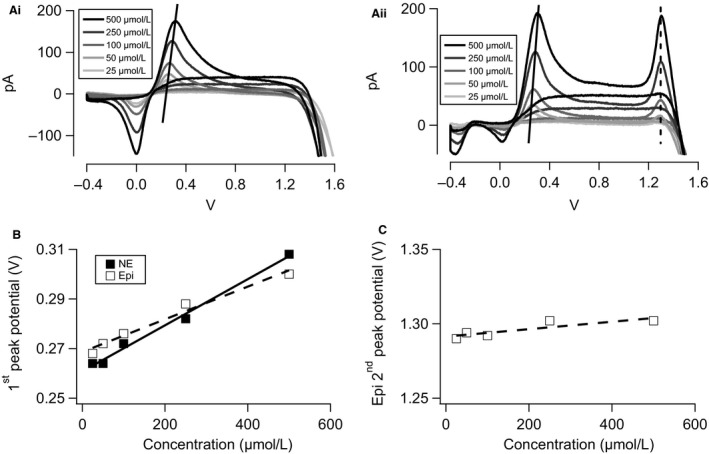
Voltage‐based calibration of the fast scanning cyclic voltammetry (FSCV) voltammograms. (Ai) The potential at which the primary oxidation reaches its peak amplitude follows a linear function (solid line) dependent on the concentration of norepinephrine (NE) in the bath and represents an independent second calibration parameter to complement the current‐based approach presented in Figure 2. (Aii) The same concentration‐dependent linear dependence on concentration observed in the NE context is also present in the primary oxidation signal for epinephrine (Epi) (solid line). No such concentration dependence is observed in the secondary oxidation signal (stippled line). (B) The concentration dependence of the potential at which the primary oxidation current reaches its peak amplitude is presented for both the NE (■, solid line) and Epi (□, stippled line). (C) The potential at which the secondary oxidation peak is reached for Epi is relatively flat at 1.3 V.

Multiple calibration parameters were obtained from voltammograms measured in standard Epi and NE solutions in vitro. The simplest parameter is the amplitude of the primary and secondary peaks in the voltammograms. Voltammograms were background subtracted as in Figure 1 and resulting current magnitudes at the primary and secondary peak potentials are plotted for NE (Fig. 2B) or Epi (Fig. 2C) against catecholamine concentrations. As expected, each peak current (primary for NE and Epi, secondary for Epi) exhibit an exponential relationship. The exponential function for the primary NE and Epi current–concentration relationships are shown as the solid lines in Figure 2B and C, while the secondary Epi current–concentration slope is shown as the dashed line in Figure 2C. In Epi solutions, a second parameter is the ratio of the second peak to the first (Fig. 2C, inset). This ratio is again dependent on Epi concentration and exhibits a reaction constant of 0.009 (*μ*mol/L)^−1^; as is expected for a higher oxidation energy in the second peak. In practice, with a FSCV recording of an unknown mixture of NE and Epi, the Epi‐specific component, if present, is defined by the amplitude of the current measured at the second oxidation potential and conversion from pA to *μ*mol/L by intersection with the calibration function (Fig. 2C). The NE‐specific component is calculated by dividing the second Epi current amplitude by the corresponding intersection of the ratio function (Fig. 2C inset) and subtracting this value from the primary oxidation current amplitude. The resulting current is then calibrated by intersection with the NE calibration function (Fig. 2B). Yet a third parameter for calibration is the observation that the potential at which the primary oxidation peak is measured shifts with catecholamine concentration. This shift is dependent on scan rate and is only readily observed at FSCV scan rates above 20 V/sec (data not shown). The shift is present for both Epi and NE (Fig. 3A–B) and is thus able to be applied to mixed catecholamine solutions and serves as a complementary measure for the amplitude measurement of the primary peak (0.093 mV/(*μ*mol/L) and 0.066 mV/(*μ*mol/L) for NE and Epi, respectively).

### Native catecholamine release from an ex vivo rat adrenal preparation

**Figure 4:**
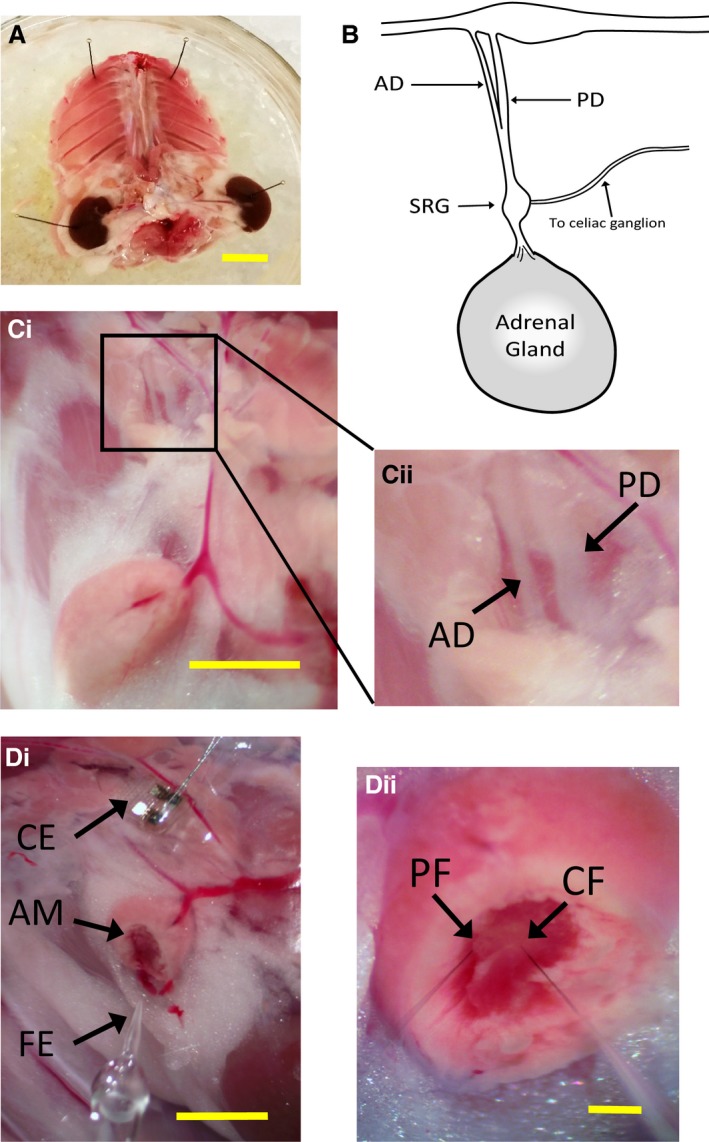
An ex vivo spinal–splanchnic–adrenal preparation. (A) Ventral view of the posterior wall of a rat is isolated between approximately T1 and L5 vertebrae. The preparation includes the entire splanchnic nerve as it innervates the adrenal gland. Scale = 10 mm. (B) A cartoon representation is provided for clarity in identifying relevant features in the ex vivo preparation. (Ci) An image similar to the cartoon representation in B is provided showing the gland in the lower region as well as the innervating splanchnic descending to the gland. Scale = 5 mm. (Cii) The inset box in Ci is blown up to show both the anterior division (AD) and posterior division (PD) of the splanchnic nerve as it innervates the adrenal gland. (Di) A cuff electrode (CE) is placed on the splanchnic nerve. The gland is hemisected to expose the medulla (AM) and an FSCV carbon fiber electrode (FE) is positioned to measure catecholamine at the exposed medulla. Scale = 5 mm. (Dii) A close‐up image of the hemisected gland shows the exposed medullary tissue which is darker in appearance. Two carbon fiber electrodes (peripheral fiber “PF” and central fiber “CF”) can be seen in the image. Scale = 1 mm.

We next set out to measure catecholamine release from the rat adrenal gland under neuronal stimulation. Toward this goal, we developed a novel ex vivo preparation. In essence, this preparation is a reduced spinal, splanchnic nerve, adrenal system maintained intact on the rear peritoneal wall of the rat (Fig. 4A, see also Materials and Methods). The preparation is bathed in Ringer as described methods and pinned out.

For clarity, we provide a cartoon representation of the splanchnic nerve as it innervates the adrenal gland in Figure 4B (see also Celler and Schramm 1981). The splanchnic nerve bifurcates as it leaves the sympathetic chain ganglion, with the anterior division (AD) smaller in diameter than the posterior division. The splanchnic passes through the super‐renal ganglion (SRG) where it gives rise to a small‐diameter fascicle that passes to the celiac ganglion, while the majority of the fibers innervate the adrenal gland. Micrographs from the actual preparation are provided in Figure 4C and D. In Figure 4Ci, the adrenal gland and super‐renal vein can be seen in the lower half of the image. The box encompasses the innervating splanchnic nerve and is blown up in panel Cii to show both the anterior division as well as the larger posterior division. Once identified, a stimulating electrode (either a platinum/iridium parallel bipolar electrode or 2‐pole cuff electrode) is placed on either the whole nerve, or either division as described below. Severing the other division served as a positive control for division‐specific stimulation. The adrenal gland is hemisected to expose the medulla and allow access to the FSCV electrodes (single electrode from the bottom in panel Fig. 4Di or dual fibers entering from below in Fig. 4Dii). This arrangement allows for division‐specific stimulation of the innervating splanchnic as well as location‐specific (peripheral versus central) measurement of secreted catecholamine (Epi and NE) from the gland.

**Figure 5:**
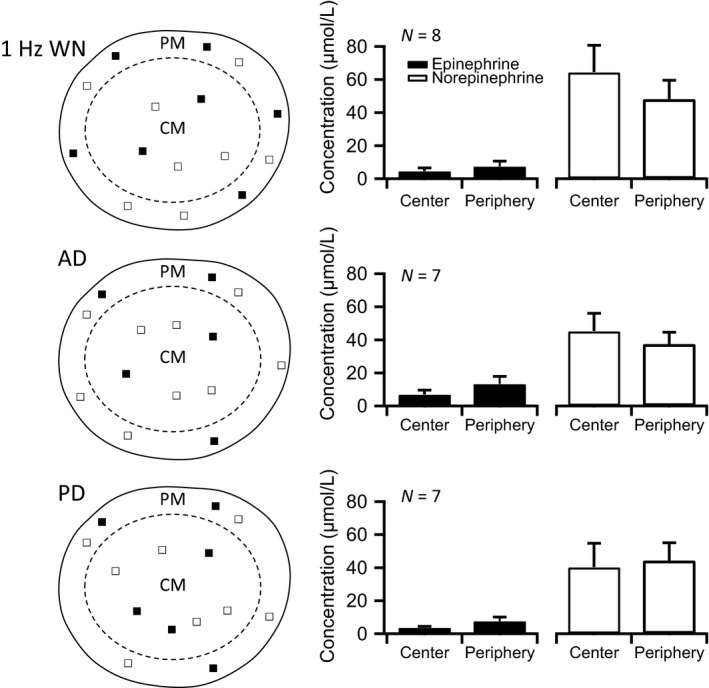
Epinephrine and norepinephrine release at 1 Hz nerve stimulation. Left column: Schematic diagram representations for the hemisected adrenal medullary face are provided. Each map is further divided into peripheral medulla (PM) and central medulla (CM) by a dotted line. Symbols demonstrate whether signal for either epinephrine (Epi) (■) or norepinephrine (NE) (□) were detected in the gland periphery or center. It should be noted that detection of both Epi and NE would provide a symbol for both. The top cartoon represents results when the whole nerve (WN) was stimulated. Below are representations for both anterior division (AD) and posterior division (PD) stimulation conditions. Right column: Epi or NE signals were calibrated as demonstrated in Figures 2 and 3 and are provided for each condition. Numbers of recordings in each dataset are provided in the upper left of each category plot. Data are supplied as mean ± SEM.

Previous reports have shown like secretory isotype chromaffin cells to be organized in groups (Vollmer 1996) that may receive common innervation (representing a functional “adrenal unit” analogous to the well‐described “motor unit” in skeletal muscle) (Feinstein et al. 1955). Moreover, specific stressors selectively elicit epinephrine versus norepinephrine release. For example, bleeding results in greater release of epinephrine relative to norepinephrine, to facilitate blood clotting and limit blood loss (Forwell and Ingram 1957; Goldstein 2010). Hypoglycemia results in elevated epinephrine release to increase hepatic blood flow as well as gluconeogenesis to elevate blood glucose levels (Vollmer et al. 1997). Conversely, cold stress results in a preferential release of norepinephrine that acts to constrict the peripheral vasculature to preserve core body heat (Vollmer 1996). Additionally, expression of either catecholamine is specific to different regions of the adrenal gland (Verhofstad et al. 1985; Ubink et al. 1995). Thus, we posed the hypothesis that specific stimulation of the anterior division versus posterior division may primarily stimulate one secretory isotype cell over the other. We tested this hypothesis by isolating either the anterior division or posterior division of the splanchnic nerve in the stimulating circuit. We then stimulated the nerve division as described and measured central versus peripheral catecholamine release from the gland. We isolated the Epi versus NE components of the catecholamine signal by the FSCV approach demonstrated in Figures 1, 2, 3. Data obtained for whole nerve, anterior division (AD) and posterior division (PD) stimulation at 1 Hz are presented in Figure 5. Positive controls included severing the other unstimulated division and negative controls were conducted where the entire nerve was cut proximal to the electrode placement. The first case provided no difference from division selection through simple electrode placement and the second case showed no Epi nor NE signal (data not shown). The left column shows a schematic of the adrenal gland and locations of detected Epi (■) versus NE (□) signal for each nerve stimulation condition. It should be noted that not all recordings provided both Epi and NE recordings in either region, in which case only one symbol was contributed to the spatial release profile. Likewise, if a recording provided both Epi and NE release within a region, both a solid and empty symbol is contributed to the release map. Thus, the maps provide a summary view of the occurrence of release for Epi and NE across recordings from either central or peripheral AM.

The right column provides quantified levels of Epi (■) versus NE (□) release at the center versus periphery of the medulla. We noted that in the whole nerve (WN) and anterior division (AD), a bias toward peripheral Epi release and central NE release was noted, although biological significance of this trend is not clear. No such bias was noted for the posterior division stimulation.

**Figure 6:**
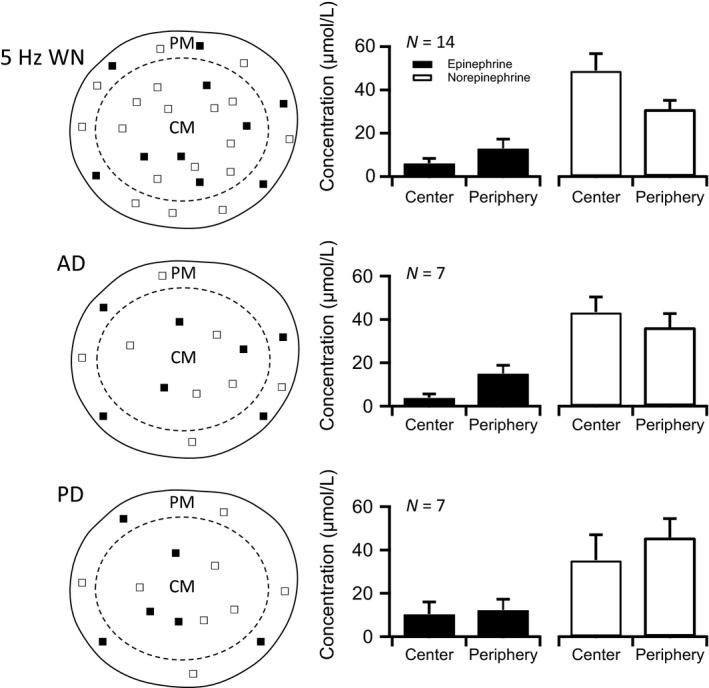
Epinephrine and norepinephrine release at 5 Hz nerve stimulation. Left column: Schematic diagram representations equivalent to those in Figure 5 are provided, except that they represent the 5 Hz stimulation condition. Symbols demonstrate whether signal for either epinephrine (Epi) (■) or norepinephrine (NE) (□) were detected in the gland periphery or center. It should be noted that a mixed signal would provide a symbol for both Epi and NE. The top cartoon represents results when the whole nerve (WN) was stimulated. Below are representations for both anterior division (AD) and posterior division (PD) stimulation conditions. Right column: Epi or NE signals were calibrated as demonstrated in Figures 2 and 3 and are provided for each condition. Numbers of recordings in each dataset are provided in the upper left of each category plot.

**Figure 7:**
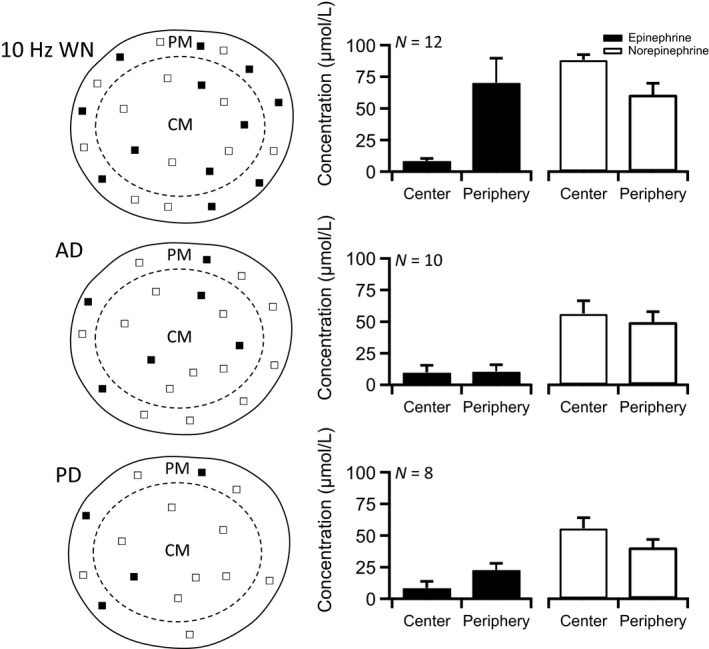
Epinephrine and norepinephrine release at 10 Hz nerve stimulation. Left column: Schematic diagram representations equivalent to those in Figure 5 are provided, except that they represent the 10‐Hz stimulation condition. Symbols demonstrate whether signal for either epinephrine (Epi) (■) or norepinephrine (NE) (□) were detected in the gland periphery or center. It should be noted that a mixed signal would provide a symbol for both Epi and NE. The top cartoon represents results when the whole nerve (WN) was stimulated. Below are representations for both anterior division (AD) and posterior division (PD) stimulation conditions. Right column: Epi or NE signals were calibrated as demonstrated in Figures 2 and 3 and are provided for each condition. Numbers of recordings in each dataset are provided in the upper left of each category plot.

Next, we repeated the same recording conditions, with the exception that nerve stimulation was at 5 Hz, a frequency chosen to approximate intermediate sympathetic activity. The resulting dataset is presented in Figure 6 and follows the same organization as that introduced in Figure 5. As in the 1 Hz condition, we noted a bias toward peripheral Epi release and central NE release. We also noted an overall increase in total catecholamine release, but no overall significant dependence on stimulation of the whole nerve versus either branch. This dependence, however, was significantly altered upon stimulation at 10 Hz (Fig. 7), a firing level chosen to mimic sympathetic activation. Under this condition, we again noted a bias toward peripheral Epi secretion and central NE release. We also noted a further increase in total catecholamine release from the adrenal gland. However, compared to previous stimulation paradigms, 10 Hz stimulation resulted in a dramatically elevated Epi release under whole nerve stimulation. Moreover, the stark elevation in peripheral Epi release was notably abolished by stimulating just one of the two divisions. Leaving either the posterior or the anterior division out of the stimulation path resulted in a failure to recruit the dramatic increase in peripheral Epi exocytosis (Table 1). Thus, it appears that while no specificity on either branch for release of Epi versus NE exists, what appears to be is that excitation of the entire nerve is required to support the surge in epinephrine release observed under heightened sympathetic firing. Previous reports have surmised the differential stimulus‐secretion behavior for NE versus Epi must be due to differential descending efferent nerve tracts (Edwards et al. 1996; Vollmer 1996). Our data indicate that by the time the splanchnic passes through the sympathetic chain ganglion, the splanchnic fibers within either division are a mixed population (Strack et al. 1988). The findings reported are novel in that they further define that Epi‐secreting cells express a higher stimulation threshold and require a larger number of active innervating fibers for maximal excitation. Once brought to threshold, Epi‐secreting cells also exhibit a steeper stimulus‐secretion function than NE‐secreting cells.

**Table 1:** Normalized peripheral catecholamine release

	1 Hz		5 Hz		10 Hz	
	Epi	NE	Epi	NE	Epi	NE
WN (8)	1 ± 0.42	1 ± 0.24	1 ± 0.32	1 ± 0.13	1 ± 0.27	1 ± 0.15
AD (7)	1.8 ± 0.61	0.78 ± 0.15	1.15 ± 0.28	1.17 ± 0.20	0.15 ± 0.07*	0.81 ± 0.12
PD (7)	1.03 ± 0.33	0.92 ± 0.22	0.94 ± 0.37	1.47 ± 0.28	0.32 ± 0.07*	0.67 ± 0.10

Peripheral epinephrine (Epi) or norepinephrine (NE) release was measured under each frequency and for each nerve stimulation condition. Each recording is from a single preparation. All values, whole nerve (WN), anterior division (AD), and posterior division (PD), were normalized internally to the WN stimulation condition to allow for comparison. Numbers of recordings for each condition (WN, AD, and PD) are supplied in parentheses. The only frequency that exhibited a statistically significant dependence on nerve fascicle integrity was Epi release at 10 Hz stimulation, with either AD‐ or PD‐specific stimulation exhibiting a significant decrease in output compared to WN stimulation. Statistical analysis compared each condition (AD, PD) for a given stimulation frequency against the matched WN control value. Significance was determined by a paired Student's *t*‐test with a barrier of *P *<* *0.05 (stared cells, *P* = 0.01 and *P* = 0.03 for 10 Hz AD and PD, respectively).

## Discussion

Under homeostatic physiological conditions, the sympathetic nervous system fires at a modest rate, setting the sympathetic tone and working in concert with the parasympathetic nervous system to place the organism into a “rest and digest” status of energy storage. Under these conditions, adrenal chromaffin cells release modest amounts of catecholamine into the circulation to help regulate physiological parameters including shunting of blood to viscera, increasing enteric activity, and maintaining basal heart rate. Emotional or psychological stress, injury or environmental insult initiates the sympathetic “fight or flight” stress response, leading to a surge in serum catecholamine levels. Under this condition, NE is released from postganglionic sympathetic nerves throughout the periphery as well as from the adrenal medulla, while Epi is exclusively released from the adrenal medulla (Marley and Prout 1965; Goldstein et al. 1983; Carmichael and Winkler 1985; Habib et al. 2001). Moreover, within the spectrum of stress responses, specific stressors differentially elevate serum levels of one catecholamine relative to the other in order to evoke the appropriate physiological counterresponse (Coupland 1958; Klevans and Gebber 1970; Goldstein et al. 1983; Scheurink and Ritter 1993; Vollmer 1996; Jeong et al. 2000; Goldstein 2010; Kvetnansky et al. 2013). For example, acute cold stress selectively elevates NE release (Vollmer 1996) to constrict peripheral vasculature in order to preserve body heat. Hemorrhage or hypoglycemia each selectively elevate Epi to stabilize blood pressure, to increase hepatic blood flow, and increase blood glucose through elevated glucagon and decreased insulin sensitivity, respectively (Glaviano et al. 1960; Gerich et al. 1973; Moyer and Mills 1975; Robertson et al. 1979; Cryer 1980; Henry 1992; Vollmer et al. 1992, 1997; Krentz et al. 1996). Other stressors evoke a broader response. Acute intermittent hypoxia (a condition found in obstructive sleep apnea patients), evokes an equivalent increase in both serum NE and Epi (Kumar et al. 2006). In this context, corelease of both catecholamines elevates pulmonary function and cardiac output to increase the supply of oxygen throughout the body. Physical restraint exhibits a more complex response, with acute immobilization initially eliciting an Epi surge, then with repeated restraint both NE and Epi are elevated (Carbonaro et al. 1988; Jeong et al. 2000). Thus, considering these differential catecholamine release responses, stressors must be able to selectively activate NE release from sympathetic terminals from Epi release that is exclusively from the adrenal medulla.

In this study, we utilized highly sensitive, fast scanning cyclic voltammetry to specifically measure Epi versus NE release from the adrenal medulla. It should be noted that the calibration method used in this dataset was intended to allow for quantitative comparison of catecholamine release across several stimulation parameters. It is clear that the concentrations presented are not analogous to those observed in serum under stress, where there is a significant dilution factor. We employed a novel ex vivo splanchnic–adrenal preparation to test for native neuronal stimulation of epinephrine versus norepinephrine in an activity‐dependent manner. We also mapped the tissue‐level organization of adrenal Epi and NE release within the gland. Using this experimental system, we tested the potential that the anterior and posterior branches of the splanchnic nerve represent a functional separation in the innervating pathway responsible for Epi versus NE release. While this was not found to be true, we did find that Epi and NE are indeed show preferentially released from different regions within the adrenal medulla. Norepinephrine release tends to occur from the central portion of the medulla, while epinephrine tends to be released from the periphery. Moreover, we find that NE release increases through a range of nerve firing rates, while Epi expresses a steep increase in release only under the highest firing rates. This steep release function is only observed under whole nerve stimulation; stimulating either branch in isolation does not express the steep increase in Epi release.

Previous work demonstrated that epinephrine‐ and norepinephrine‐secreting cells are innervated by calretinin‐negative and ‐positive fibers, respectively, and that calretinin‐positive fibers are predominant in the rostral portion of the spinal cord (Edwards et al. 1996). Due to its relative caudal position, it would be expected that the anterior division of the splanchnic may include a lower proportion of calretinin‐positive nerve fibers, and thus preferentially stimulate epinephrine‐secreting chromaffin cells. This was not observed, indicating that these specific fiber tracts mix and lose anatomic organization prior to or as the nerve exits the spinal cord. It may be that neurons in the spinal cord are activated in a stressor‐specific manner from various central control circuits (Strack et al. 1988; Cao and Morrison 2001), integrate them, and output a signal determined by activation of specific calretinin‐positive or ‐negative nerve fascicles, innervating specific patches of cells in the adrenal medulla. In this way, selective catecholamine release could be determined, with whole adrenal units (clusters of like isotype chromaffin cells) modulated by paracrine effects of catecholamines (Kajiwara et al. 1997; Brede et al. 2003) and potentially neuropeptide release (Aunis 1998).

Thus, it seems that the splanchnic nerve does not follow an anatomical organization with respect to the branches. Each branch must be a mixed population of NE‐ and Epi‐innervating fibers. Moreover, the data provided here demonstrate that the higher stimulus threshold for Epi versus NE secretion follows a simple capacity function; it does not matter which division of the splanchnic is stimulated or cut, maximal recruitment of Epi secretion capacity is lost by eliminating splanchnic nerve fibers, no matter their location. Maximal Epi section is only achieved with all possible nerve fibers participating. The surge in epinephrine shown under the sympathoadrenal stress reflex is due to a higher threshold, steeper stimulus‐secretion function than that for norepinephrine.

Adrenal chromaffin cells are polyinnervated, receiving between 1 and 4 synaptic contacts each. It is not known whether this heterogeneity in polyinnervation correlates with cell isotype. Previous studies have shown that Epi‐ and NE‐secreting chromaffin cells have different numbers of synapses (Iijima et al. 1992; Kajiwara et al. 1997), which may provide an additional potential explanation for our observations. If NE‐secreting cells are preferentially innervated by more splanchnic terminals than Epi‐secreting cells, they may be expected to exhibit catecholamine release under modest splanchnic stimulation due to simultaneous excitatory inputs. Likewise, only after more intense splanchnic firing, are the Epi‐secreting cells brought to threshold for excitation and secretion. This potential model will require further testing through histological and electrophysiological investigation of synapse number and synaptic excitation in chromaffin cells. Finally, accessory transmitters other than acetylcholine are released from the splanchnic terminals that act as strong secretagogues for chromaffin cell catecholamine release. Pituitary adenylyl cyclase activating peptide (PACAP) is released specifically under elevated sympathetic firing and evokes the stress‐associated surge in adrenal catecholamine release (Hamelink et al. 2002; Kuri et al. 2009; Smith and Eiden 2012). Future experiments will need to address this point. It may be that splanchnic efferents innervating Epi‐ versus NE‐secreting cells express different levels of PACAP or that Epi‐ versus NE‐secreting cells exhibit differential sensitivity to splanchnic PACAP release through receptor expression. These possible mechanisms for the differing stimulus–secretion relationship in Epi‐ versus NE‐secreting cells also will require significant investigation for determination of the potential molecular basis of stressor‐specific catecholamine release.

## Conflict of Interest

Case Western Reserve University and GlaxoSmithKline have filed an Invention Disclosure on the process for modulating epinephrine release through splanchnic stimulation.

## Supporting information




**Figure S1.** In vitro calibration measured as integrated current. Voltammograms were measured in standard concentrations of either norepinephrine (NE) or epinephrine (Epi), background subtracted, and plotted as described in the text for Figure 2. Catecholamine‐specific currents were integrated to provide total detected charge and are plotted against catecholamine concentration. (A) The integral of the first peak in the NE calibration voltammogram set follows an exponential function depending on NE concentration. (Bi) Both the primary and secondary oxidation current integrals for Epi follow exponential dependencies on concentration as well. (Bii) As observed for the peak current versus epinephrine concentration plotted in Figure 2 of the manuscript, the ratio of the second peak integral to the first follows an exponential function, in this case with a reaction constant of 0.014 *μ*mol/L.
**Table S1.** Raw catecholamine values for all conditions (*μ*mol/L).
