# Supplementary material for: Spatial and activity‐dependent catecholamine release in rat adrenal medulla under native neuronal stimulation
Source: Physiol Rep. 2016 Sep 5;4(17):e12898. doi: 10.14814/phy2.12898 (PMC5027341; doi:10.14814/phy2.12898)
Supplement: Supplementary file 1 — Figure S1. In vitro calibration measured as integrated current. Voltammograms were measured in standard concentrations of either norepinephrine (NE) or epinephrine (Epi), background subtracted, and plotted as described in the text for Figure 2. Catecholamine‐specific currents were integrated to provide total detected charge and are plotted against catecholamine concentration. (A) The integral of the first peak in the NE calibration voltammogram set follows an exponential function depending on NE concentration. (Bi) Both the primary and secondary oxidation current integrals for Epi follow exponential dependencies on concentration as well. (Bii) As observed for the peak current versus epinephrine concentration plotted in Figure 2 of the manuscript, the ratio of the second peak integral to the first follows an exponential function, in this case with a reaction constant of 0.014 μmol/L. Table S1. Raw catecholamine values for all conditions (μmol/L). [file PHY2-4-12898-s001.docx]

**Supplemental Figure 1. In vitro calibration measured as integrated current.**

Voltammograms were measured in standard concentrations of either norepinephrine (NE) or epinephrine (Epi), background subtracted and plotted as described in the text for figure 2. Catecholamine specific currents were integrated to provide total detected charge and are plotted against catecholamine concentration. **(A)** The integral of the first peak in the NE calibration voltammogram set follows an exponential function depending on NE concentration. **(Bi)** Both the primary and secondary oxidation current integrals for Epi follow exponential dependences on concentration as well. **(Bii)** As observed for the peak current versus epinephrine concentration plotted in Figure 2 of the manuscript, the ratio of the second peak integral to the first follows an exponential function; in this case with a reaction constant of 0.014 µM^-1^.

| **Supplementary table 1. Raw Catecholamine Values for all Conditions (µM).** | | | | |
| --- | --- | --- | --- | --- |
|  |  |  | | |
|  |  | Epinephrine | | |
| Nerve |  | Whole | Anterior | Posterior |
| Center | 1 Hz | 4.59 ± 1.93 (8) | 6.89 ± 2.71 (7) | 3.62 ± 0.8 (7) |
|  | 5 Hz | 6.22 ± 2.13 (14) | 3.91 ± 1.69 (7) | 10.49 ± 5.56 (7) |
|  | 10 Hz | 8.56 ± 1.82 (12) | 9.85 ± 5.56 (10) | 8.57 ± 5.23 (8) |
|  | | | | |
| Peripheral | 1 Hz | 7.41 ± 3.16 (8) | 13.33 ± 4.53 (7) | 7.64 ± 2.44 (7) |
|  | 5 Hz | 13.09 ± 4.20 (14) | 15.11 ± 3.71 (7) | 12.36 ± 4.91 (7) |
|  | 10 Hz | 70.29 ± 19.27 (12) | 10.67 ± 5.26 (10) | 22.86 ± 5.08 (8) |
|  | | | | |
|  |  | Norepinephrine |  |  |
| Nerve |  | Whole | Anterior | Posterior |
| Center | 1 Hz | 64.46 ± 16.20 (8) | 45.28 ± 10.76 (7) | 40.38 ± 14.40 (7) |
|  | 5 Hz | 48.96 ± 7.78 (14) | 43.36 ± 6.97 (7) | 35.31 ± 11.72 (7) |
|  | 10 Hz | 88.32 ± 4.10 (12) | 56.31 ± 10.11 (10) | 55.76 ± 8.25 (8) |
|  |  |  |  |  |
| Peripheral | 1 Hz | 48.15 ± 11.35 (8) | 37.5 ± 7.14 (7) | 44.29 ± 10.75 (7) |
|  | 5 Hz | 31.12 ± 4.00 (14) | 36.42 ± 6.29 (7) | 45.78 ± 8.69 (7) |
|  | 10 Hz | 60.76 ± 9.04 (12) | 49.72 ± 8.15 (10) | 40.67 ± 6.13 (8) |

**Supplementary table 1.** Numeric values for all stimulation conditions are provided. Data are supplied as mean measured catecholamine detected (in µM) ± S.E.M. Numbers of recordings for each condition are supplied in parentheses.
